# Supplementary material for: The Influence of Individual and Contextual Factors on the Vocational Choices of Adolescents and Their Impact on Well-Being
Source: Behav Sci (Basel). 2023 Mar 7;13(3):233. doi: 10.3390/bs13030233 (PMC10045217; doi:10.3390/bs13030233)
Supplement: Supplementary file 1 [file behavsci-13-00233-s001.zip › behavsci-2185744-supplementary.pdf]

Table S1. Skewness and Kurtosis values for the hypothesised predictors and outcomes (N=147)

Variable	Skewness	Kurtosis
MCC-C	-.807	-.285
PCC-C	-.729	-.365
MCC-S	-.078	-.832
PCC-S	-.162	-.930
WH-A	-.705	.311
WH-P	-.113	-.278
WH-G	-.570	.007
AM-A	2.000	3.657
AM-E	-.284	-.844
AM-IO	.383	-.902
AM-ID	-1.29	1.037
AM-II	-.476	-.279
SM-FV	-.84	.843
IM-FV	-1.162	1.273
OM-FV	-1.195	1.428
CM-FV	-.815	-.055
SM-AV	-1.167	1.686
IM-AV	-1.775	3.378
OM-AV	-1.1	.690
CM-AV	-.852	.276
INT-UN	-1.635	2.634
I WB	-.740	0.023
C WB	-.974	1.493
O WB	-1.786	3.066
PH WB	-.953	.650
PS WB	-.849	.665
E WB	-1.029	1.633
OV WB	-1.378	2.742
